# Pain on the first postoperative day after tonsillectomy in adults: A comparison of metamizole versus etoricoxib as baseline analgesic

**DOI:** 10.1371/journal.pone.0221188

**Published:** 2019-08-14

**Authors:** Katharina Geißler, Marina Ducke, Gerd Fabian Volk, Winfried Meißner, Orlando Guntinas-Lichius

**Affiliations:** 1 Department of Otorhinolaryngology, Jena University Hospital, Jena, Germany; 2 Department of Anaesthesiology and Intensive Care Medicine, Jena University Hospital, Jena, Germany; Cleveland Clinic, UNITED STATES

## Abstract

**Objective:**

To compare the effect of metamizole versus etoricoxib as baseline analgesic for treating postoperative pain after tonsillectomy.

**Design:**

Single centre prospective cohort study.

**Setting:**

Two consecutive cohorts of tonsillectomy patients.

**Participants:**

124 patients (n = 55 treated with etoricoxib, n = 69 with metamizole); median age 30.5 years; 50% women.

**Main outcome measures:**

Patients rated their pain on first postoperative day using the questionnaires of the German-wide project Quality Improvement in Postoperative Pain Treatment (QUIPS) including numeric rating scales (NRS, 0–10) for pain determination. The influence of preoperative and postoperative parameters on patients' pain was estimated by univariate and multivariate statistical analysis.

**Results:**

The demographic parameters showed no differences between the patients in the metamizole group and the etoricoxib group (all p>0.05) with one exception: Patients in the metamizole group had significantly more preoperative pain than patients in the etoricoxib group (p = 0.001). The metamizole group had a mean postoperative pain in activity of 4.4 ± 2.1 and the etoricoxib group of 4.5 ± 2.2. Maximal pain for the metamizole group and the etoricoxib group were 5.6 ± 2.2 and 6.1 ± 1.9, respectively. Pain in activity, maximal pain and minimal pain were not different between both groups (p = 0.652, p = 0.113, p = 0.276, respectively). Patients of the etoricoxib group received more frequently piritramide in recovery room as demand medication (p = 0.046). In the whole cohort, patients with peritonsillar abscess had more preoperative pain in comparison to chronic tonsillitis (p<0.001). Patients under 30.5 years reported higher maximal pain than older patients (p = 0.049). On the other hand, a significant influence of patients’ age on the pain in activity and minimal pain could not be demonstrated (p = 0.368, p = 0.508, respectively). Men reported lower minimal pain than women (p = 0.041). Also, patients with ASA status I had lower minimal pain than patients with higher ASA status (p = 0.019). The multivariate analysis did not show an association between postoperative pain in activity and preoperative counseling on postoperative pain management (p = 0.588, p = 0.174, respectively). Special preoperative counseling on postoperative pain management resulted in lower levels of maximal pain (p = 0.024). Linear regression demonstrated an independent association of higher pain in activity with higher mobility impairment (p = 0.034) and respiratory impairment (p = 0.002). The linear regression of minimal pain identified female gender (p = 0.005) as an independent influencing factor with higher pain levels. In terms of satisfaction, no preoperative pain therapy (p = 0.016) could be found as an independently significant influencing factor with higher satisfaction.

**Conclusion:**

Etoricoxib does not have an advantage as baseline analgesic for post tonsillectomy pain in comparison to metamizole.

## Introduction

Tonsillectomy, the surgical removal of the palatine tonsils, is still one of the most common surgical procedures in adults. For instance, 54.441 tonsillectomies were performed in Germany in 2014 [1]. Tonsillectomy causes severe postoperative pain lasting for many days [2]. A prospective cohort study taking part in the Quality Improvement in Postoperative Pain Treatment (QUIPS) registry has shown that tonsillectomy was one of the most painful surgical procedures even compared to major surgery procedures [3]. QUIPS was developed in 2005, consisting of standardized data acquisition and an analysis of quality and process indicators [4]. QUIPS and the international counterpart PAIN OUT are open for every hospital worldwide and are web-based [5, 6, 7]. Recent studies confirmed that postoperative pain is relevant after tonsillectomy but pain management may be insufficient and needs improvement [5, 8, 9]. There is no international standard pain therapy regime for adults after tonsillectomy.

For pain therapy after tonsillectomy typically a combination of a non-opioid as basic analgesic with an opioid is used in adults, e.g. a non-steroid anti-inflammatory drug (NSAID) like metamizole and more recently, also cyclooxygenase (COX)-2-inhibitors are combined with an opioid like piritramide or tramadol.

The present prospective clinical study used QUIPS data to analyse 1) if the COX-2-inhibitor etoricoxib 90mg per os once a day in the morning as basic analgesic after tonsillectomy reduces postoperative pain better than metamizole 1g intravenous four times a day every six hours, 2) the quality of pain management after tonsillectomy, and 3) other factors that influence postoperative pain.

## Methods

The present prospective cohort study was part of the German-QUIPS registry. Institutional review board approval was obtained prior to study initiation by the Ethics Committee of the Jena University Hospital, Thuringia, Germany. The patients gave written consent.

### Subjects

Patients treated in the hospital as in-patients between November 2008 and May 2009 (metamizole cohort) and February 2013 and December 2013 (etoricoxib cohort) were included. By German law, the German procedure classification (OPS-301) has to be used by all German hospitals for coding of surgical procedures (OPS codes). To select all patients with tonsillectomy, first all patients with the OPS codes 5–281.0 (tonsillectomy without adenoidectomy) and 5–281.1 (tonsillectomy for peritonsillar abscess) were selected. 124 of 189 adult patients were included. 55 patients were excluded because they refused to participate, and 5 patients because of a lack of knowledge of the German language. One patient was in bad physical conditions and not able for performing the questionnaires. In two cases the histopathological examinations revealed a malignant tumor. In two patients the medication for pain management was not following the protocol and therefore they were excluded.

### Pain and pain management measures

The QUIPS questionnaires are presented in detail elsewhere [9]. The QUIPS questionnaires consists of two parts for each patient: This first part is covering the outcome parameters of the questionnaire, whereas the second part is filled by the investigator. After a standardized instruction by a PhD student or attending nurses, the patient her-/himself completed the part one of the form. The patients received the validated 15-item QUIPS questionnaire at the first postoperative day. QUIPS uses 11-point numeric rating scales (NRS) to estimate the patient’s pain during activities, maximal pain and pain at rest. Generally, higher numbers are indicating more pain (0 = no pain; 10 = maximal pain). Furthermore, the patient was asked by dichotomized (yes/no) questions about pain-related impairments (mobility, breathing, sleep, mood), side effects of pain treatment (drowsiness, nausea, vomiting), and satisfaction with the pain management. The patients were also asked about the preoperative pain counselling in three categories (yes, in general; yes, specific; no). General pain counselling meant that education about postoperative pain and its management in general was part of the pre-surgical interview with the patients. Specific pain counselling assumed that it was talked about specific pain related to the surgical procedure the patient underwent. Furthermore, the interview had to include education on specific measures to prevent and manage postoperative pain before, during and after tonsillectomy exactly for the interviewed patient. The second part, which is filled by the investigator, was covering the relevant demographic and clinical parameters like age, gender, type of surgery, anaesthesia, and pain management. The question of equipotency of etoricoxib 90mg per os once a day in the morning and metamizole 1g intervenous four times a day every six hours is difficult to answer as there are no head-to-head comparisons. According to Moore et al. and our clinical experience, both drugs seem to be relatively equipotent [10].

### Statistical analysis

The statistical analysis was performed with IBM SPSS statistics software (Version 23.0.0.0). Data are presented as mean ± standard deviation (SD) if not otherwise indicated. Clinical and outcome parameters of all patients were summarized descriptively. To compare the metric data of two independent groups the nonparametric Mann-Whitney-U-test was used. If there were variables with more than 2 possible answers, the nonparametric Kruskal-Wallis-ANOVA-test was used. To check for significance in nominal variables, Pearson`s chi-square test was applied. The significance level was set at p<0.05. For the comparison of dependent variables, the nonparametric Wilcoxon-test was used. For correction of multiple testing, the Bonferroni method was applied. Multivariable binary logistic regression models with stepwise entry were used for the dichotomized categorized outcome parameters to analyse the association to pain-related interferes and pain therapy side effects. Patients’ characteristics and clinical parameters for regression analysis were derived from those suggestive for significant associations from the univariate analyses (p<0.05). In general, nominal p values of two-tailed tests are reported.

## Results

### Demographic parameters

Patients`characteristics are documented in Table 1. The characteristics were not different between the patients in the metamizole group and the etoricoxib group (all p>0.05). No major postoperative bleeding needing an intervention occurred in both groups.

**Table 1:** Baseline characteristics of the patients in both groups.

Parameter	All patients n = 124	Metamizole group n = 69	Etoricoxib group n = 55	p-value
Gender				
Female	62	33	29	0.547
Male	62	36	26	
Diagnosis				
Chronic tonsillitis	76	35	43	0.257
Peritonsillar abscess	48	34	12	
CRP				
< 4.6 mg/l (median)	56	24	32	0.106
≥ 4.6 mg/l (median)	55	32	23	
	Mean ± SD	Mean ± SD	Mean ± SD	
Age in years	33 ± 12	36 ± 13	31 ± 10	0.061

ASA = American Society of Anesthesiologists, CRP = C-reactive protein, SD = standard deviation.

### Postoperative pain

Table 2 gives a detailed overview over preoperative, intraoperative and postoperative received medication on first postoperative day in both treatment groups. Patients in the etoricoxib group received significantly more often midazolam as premedication (p = 0.001) and remifentanil intraoperatively (p = 0.001). Patients of the etoricoxib group received significantly more piritramide in recovery room (p = 0.046).

**Table 2:** Preoperative, intraoperative and postoperative received medication on first postoperative day.

Parameter	All patients	Metamizole group n = 69	Etoricoxib group n = 55	p-value
counseling about pain therapy				0.960
general	93	52	41	
special	25	14	11	
not	6	3	3	
premedication				0.001
midazolam	113	58	55	
no sedatives	10	10	0	
missing data	1	1	0	
intraoperative medication				0.001
remifentanil	69	29	40	
clonidine	16	15	1	
postoperative medication in recovery room and on ward				
piritramide in recovery room				0.046
yes	58	27	31	
no	66	42	24	
metamizole in recovery room				<0.001
yes	51	51	0	
no	73	18	55	
acetaminophen in recovery room				0.639
yes	2	2	0	
no	122	67	55	
need of analgetics in recovery room				0.078
yes	32	9	23	
no	92	60	32	
pethidine in recovery room				0.843
yes	1	0	1	
no	123	69	54	
metamizole on ward				0.001
yes	51	51	0	
no	73	18	55	
acetaminophen on ward				0.911
yes	1	1	0	
no	123	68	55	
no baseline NSAID on ward				0.749
yes	2	2	0	
no	122	67	55	
etoricoxib on ward				0.001
yes	55	0	55	
no	69	69	0	
additional piritramide				0.001
yes	51	6	45	
no	73	63	10	
additional ibuprofen				0.983
yes	5	1	4	
no	119	68	51	
additional tramadol				0.521
yes	2	2	0	
no	122	67	55	
additional acetaminophen				0.489
yes	1	1	0	
no	123	68	55	
additional metamizole				0.001
yes	27	0	27	
no	97	69	28	
postoperative antibiotics	81	41	40	0.342
yes	81	41	40	
no	43	28	15	
chronic tonsillitis				<0.001
yes	37			
no	39			
peritonsillar abscess				<0.001
yes	44			
no	4			
	Mean ± SD	Mean ± SD	Mean ± SD	
pain during activity	4.5 ± 2.1	4.4 ± 2.1	4.5 ± 2.2	0.652
maximal pain	5.8 ± 2.1	5.6 ± 2.2	6.1 ± 1.9	0.113
minimal pain	2.3 ± 1.6	2.2 ± 1.6	2.4 ± 1.7	0.276

SD = standard deviation

In Table 3 important differences between the group of metamizole and etoricoxib according to chronic pain were presented. Patients in the metamizole group had significantly more preoperative pain than patients in group of etoricoxib (p = 0.001). Patients with peritonsillar abscess, had more preoperative pain than patients with chronic tonsillitis (p<0.001).

**Table 3:** Presentation of chronic pain and postoperative pain-associated and pain therapy-associated impairments.

Parameter	All patients	Metamizole group n = 69	Etoricoxib group n = 55	p-value
chronic pain				
preoperative	6.1 ± 3.0	7.2 ± 2.2	4.2 ± 1.9	0.001
chronic tonsillitis	4.3 ± 1.9			<0.001
peritonsillar abscess	8.1 ± 1.5			
postoperative pain-associated and pain therapy-associated impairments				
pain while breathing				0.306
yes	87	51	36	
no	37	18	19	
waking up because of pain				0.087
yes	66	32	34	
no	58	37	21	
fatigue				0.675
yes	55	32	23	
no	69	37	32	
feeling uncomfortable because of pain				0.871
yes	37	21	16	
no	87	48	39	
complaining by mobility restricts				0.958
yes	16	9	7	
no	108	60	48	
nausea				0.483
yes	7	3	4	
no	117	66	51	
vomitus				0.817
yes	4	2	2	
no	120	67	53	
desire for pain killers				0.548
yes	20	10	10	
no	104	59	45	
satisfaction with pain therapy	11.6±3.2	11.5±3.4	11.7±3.0	0.916

### Univariate and multivariate analysis of factors associated to postoperative pain

#### Factors associated to postoperative pain

There was a significant correlation between the age of the patients and the maximal pain. Patients younger than 30.5 years reported significantly higher maximal pain than older patients (p = 0.049). A significant influence of patients’ age on the stress pain and minimal pain could not be demonstrated (p = 0.368, p = 0.508, respectively). Men reported lower minimal pain than women (p = 0.041). Patients with ASA status I had significantly lower minimal pain than patients who belonged to ASA groups II and III (p = 0.019). For pain in activity, maximal pain and minimal pain there were no significant differences between metamizole and etoricoxib group. The results of the univariate analyses on factors associated to postoperative pain are summarized in **S1, S2** and **S3 Tables**.

#### Factors associated to pain-related and pain-treatment related symptoms

Younger patients felt more affected by pain in their mood than older patients in the group (p = 0.011; S4 Table). In addition, there was a significant association between age and the desire for more analgesics, with the subgroup below the median more often expressing a desire for more analgesics. There are no significance differences between metamizole and etoricoxib group according to questions E5 to E12. Patients diagnosed with chronic tonsillitis awoke more frequently at night (p = 0.016) and were more likely to experience nausea (p = 0.042). When a patient was operated as an emergency case, he awoke significantly less frequently at night (p = 0.007). Significant association was also found between ASA status and respiratory distress and nausea after surgery: patients with ASA status 1 had significantly less respiratory distress than patients with higher ASA status (p = 0.008). Patients with CRP below the median of 4.6 mg/l were more likely to experience more pain at night than patients with higher CRP (p = 0.018).

#### Influencing factors for satisfaction with pain therapy

Examination of the demographic parameter for its impact on patient satisfaction with pain treatment showed significantly higher satisfaction levels within the patient group above the median age (p = 0.006). The diagnosis of chronic tonsillitis was associated with lower satisfaction scores; Patients with a diagnosis of peritonsillar abscess, on the other hand, were significantly more satisfied with pain management (p = 0.002). Detailed values can be found in **S5 Table.**

#### Influence of process parameters on postoperative pain

Patients who received opioids in the recovery room reported significantly higher maximal pain than those in the recovery room without opioids (p = 0.011). The same was observed with the administration of opioids on ward (p < 0.001). This was also evident with regard to pain in activity (p = 0.001). When a patient received metamizole in the recovery room, he also reported higher minimal pain (p = 0.049). In contrast to tramadol and tilidine, patients receiving piritramide had significantly more pain in activity and maximum pain than patients who did not receive piritramide (p = 0.005, p = 0.001). The choice of baseline pain medication on ward had no significant effect on postoperative pain intensity: pain in activation, maximal pain and minimal pain were not significantly lower in the etoricoxib group than in the group receiving metamizole (p = 0.841, p = 0.267, p = 0.994, respectively). Also, there was no significantly lower pain in the etoricoxib group in those patients who had already received etoricoxib preoperatively as premedication (p = 0.423, p = 0.474, p = 0.286, respectively). Already preoperative pain therapy was associated with higher pain in activity (p = 0.003) and maximal pain (p = 0.020). In addition, significant correlations between pain counseling and the pain intensity were found: Patients informed about special pain therapy procedures reported lower pain in activity and maximal pain (p = 0.004, p = 0.009, respectively). Detailed values can be found in **S6, S7** and **S8 Tables.** The counseling was performed by the same physician who explained the surgery. The general counseling included the fact that tonsillectomy will lead to pain. This pain will be treated by pain medication. The detailed counseling included information about the difference of baseline and PRN medication. The patients were asked not to hesitate to ask for PRN medication. It was explained how important it is that the patient does not reach pain peaks as late as before asking for pain medication. The short-term use of opioids was explained in detail.

#### Multivariate analysis of the association between influencing factors and postoperative pain

No significance association could be found between the pain in activity and preoperative counseling on postoperative pain management (p = 0.588, p = 0.174, respectively). Special preoperative counseling on postoperative pain management resulted in lower maximal pain (p = 0.024).

Linear regression demonstrated higher mobility impairment (p = 0.034) and higher respiratory impairment (p = 0.002) as independent factors for more pain in activity; that means, patients with more pain received more opioids, so that the increased administration of opioids could be related to respiratory impairment and mobility impairment because of respiratory impairment.

Female Gender (p = 0.005) was an independent influencing factor for more minimal pain. In terms of satisfaction with pain therapy, only the preoperative pain therapy (p = 0.016) could be found as an independently influencing factor with lower satisfaction in case of preoperative pain therapy. Details on the multivariate analyses can be found in **Table 4**.

**Table 4:** Binary logistic regression analysis on independent influence factors on pain parameters.

pain in activity R^2^ = 0.205, p<0.001	beta	95% CI lower limit	95% CI upper limit	standardized beta	p-value
mobility impairment (yes = 1, no = 0)	1.107	0.084	2.130	0.178	**0.034**
respiratory impairment (yes = 1, no = 0)	1.230	0.466	1.993	0.269	**0.002**
maximal pain R^2^ = 0.192, p = 0.174					
specific pain medication counseling (yes = 1, no = 0)	-3.169	-5.867	-0.471	-0.618	**0.024**
minimal pain R^2^ = 0.141, p = 0.001					
gender (female = 1, male = 0)	0.783	0.247	1.318	0.243	**0.003**
satisfaction R^2^ = 0.092, p = 0.008					
preoperative pain therapy (yes = 0, no = 1)	-1.776	-1.387	-0.341	-2.451	**0.016**

## Discussion

Although tonsillectomy is known to be one of the most painful surgeries[3], there is still no standard of pain therapy leading to sufficient analgesia in all tonsillectomy patients. The present study evaluated the effect of a baseline pain therapy with a newer type of NSAID, the cylcooxygenase (COX)-2-inhibitor etoricoxib versus metamizole. In conclusion, the administration of etoricoxib as a basic analgesic did not improve the level of pain in activity, maximal pain or minimal pain when compared directly to metamizole. Younger patients under 30.5 years reported higher maximal pain than older patients. Men reported significantly lower minimal pain than women. Special preoperative counseling on postoperative pain management resulted in lower levels of maximal pain.

Strengths of this study are the large sample size and the standardized assessment of process and outcome parameters with QUIPS [8]. The weaknesses of this study were the lack of randomization and the measurement only on the first postoperative day. Furthermore, metamizole is not licensed for acute pain treatment in adults in many countries. The presented results are only relevant for those countries where a metamizole treatment is possible.

One should also be aware that COX-2-inhibitors have a specific safety profile. Due to a higher risk of cardiovascular risks compared to NSAIDs [11], the application in older patients (not the typical age group for tonsillectomy) should be weighted with caution.

Younger patients under 30.5 years reported higher maximal pain than older patients. Patients younger than 50 years were significantly more affected in their mood than patients who were older. The literature often reports a lower level of pain experienced by older patients compared to the younger ones [12, 13], however this could only be found in a lower analgesic consumption of older patients. This could be explained by an age-dependent conversion of the metabolism and thus pharmacokinetics and pharmacodynamic, which leads to an increased sensitivity and prolonged effect of opioids [14]. On the other side, there are also a number of studies that could find no difference in pain perception between the age groups [15].

For pain therapy in the recovery room 41% of patients received a non-opioid (mainly metamizole), while 48% of patients received the opioid piritramide. There was a significant difference between the etoricoxib group and the metamizole group in 39%. The PRN administration of significantly more piritramide in the group of etoricoxib could be part of the conclusion that metamizole showed better analgetic results.

The positive correlation between maximal pain and administration of opioids in the recovery room and between minimal pain and administration of metamizole in the recovery room found in the univariate analysis could not be confirmed as independent factors in the multivariate analysis. In contrast, Inhestern et al., also using the QUIPS tool, showed a correlation between the administration of opioids and non-opioids and the pain or pain-related impairments on the first postoperative day [16]. Inhestern et al. analyzed not only patients with tonsillectomy. They included many other types of head and neck surgery, which may explain the differences in the results.

Despite important pain levels, the satisfaction with the pain therapy was quite high in both groups. Such a paradox effect has been described in numerous studies [17, 18, 19]. This phenomenon is explained in the literature by the patient's expectation of a certain degree of pain or the low demands on pain therapy [20, 21, 22]. More than pain intensity the perceived impression of the clinician's efforts to relieve patient pain and well-being seems to have an impact on satisfaction [22, 23, 24]. Finally, the patient's anxiety may also play a role due to a negative evaluation that negatively influences further treatment [25].

Mood impairment and the desire for more painkillers were factors influencing satisfaction with pain therapy. A negative influence of pain in activity or maximum pain on satisfaction found in the analysis could not be confirmed as independent in the multivariate analysis.

It has been already shown that preoperative counseling of patients can lead to a significant reduction of postoperative pain. Two recent studies were able to demonstrate a positive influence of the preoperative special education about the pain therapy on pain parameters, postoperative impairments and the satisfaction of the patients by means of QUIPS[8, 26]. A targeted and detailed education of the patient about the pain therapy after the operation can strengthen his confidence in the clinic and reduce fears. Thus, Papanastassiou et al. showed an anxiety reduction and greater satisfaction with pain therapy following preoperative patient education [27]. Lin and Wang showed lower pain intensities [28]. We could confirm this effect. Lower maximal pain levels were seen in patients who had been specially informed preoperatively. Therefore, a preoperative patient education about postoperative pain management should become a standard for all patients prior to tonsillectomy.

It will be necessary to continue to search for suitable painkillers or pain therapy concepts and to test their effectiveness with the help of QUIPS. Maybe a combination of diclofenac, etoricoxib or metamizole with oxycodone or a combination of tilidine and naloxone is conceivable. In the future pain should be asked standardized daily from the first postoperative day to the last day in hospital. After each pain therapy the pain intensity should be collected and compared with the success of therapy. The preoperative counseling of patients about pain therapy should be an integral part of conservation between doctor and patient because it could have a positive effect on postoperative pain.

## Conclusion

The present study on 124 adult tonsillectomy patients showed average pain values above the intervention limit of 4 on the NRS despite standardized pain therapy. Optimal post-tonsillectomy analgesia remains unclear. The administration of etoricoxib as a basic analgesic did not improve the level of pain when compared directly to metamizole. It will be necessary to continue to search for suitable painkillers or pain therapy concepts and to test their effectiveness with the help of QUIPS. Maybe a combination of diclofenac, etoricoxib or metamizole with oxycodone or a combination of tilidine and naloxone is conceivable. In the future pain should be asked standardized daily from the first postoperative day to the last day in hospital. After each pain therapy the pain intensity should be collected and compared with the success of therapy. The preoperative counseling of patients about pain therapy should be an integral part of conservation between doctor and patient because it could have a positive effect on postoperative pain.

## Supporting information

S1 Table Influence of demographic parameters on pain in activity. (DOCX)

S2 Table Influence of demographic parameters on maximum pain. (DOCX)

S3 Table Influence of demographic parameters on minimum pain. (DOCX)

S4 Table Influence of demographic parameter on postoperative pain-associated and pain therapy-associated impairments. (DOCX)

S5 Table Influence of demographic parameters on the satisfaction with pain therapy. (DOCX)

S6 Table Influence of process parameter on pain in activity. (DOCX)

S7 Table Influence of process parameter on maximum pain. (DOCX)

S8 Table Influence of process parameter on minimum pain. (DOCX)
