# Supplementary material for: Pain on the first postoperative day after tonsillectomy in adults: A comparison of metamizole versus etoricoxib as baseline analgesic
Source: PLoS One. 2019 Aug 14;14(8):e0221188. doi: 10.1371/journal.pone.0221188 (PMC6693748; doi:10.1371/journal.pone.0221188)
Supplement: S1 Table — (DOCX) [file pone.0221188.s001.docx]

**S1 Table** Influence of demographic parameters on pain in activity

| Parameter | Mean ± SD | p-value |
| --- | --- | --- |
| pain in activity | 4.5 ± 2.1 |  |
| age |  | 0.368 |
| ≤median | 4.3 ± 2.2 |  |
| >median | 4.6 ± 2.0 |  |
| gender |  | 0.590 |
| female | 4.4 ± 2.2 |  |
| male | 4.6 ± 2.0 |  |
| diagnosis |  | 0.091 |
| chronic tonsillitis | 4.5 ± 2.1 |  |
| peritonsillar abscess | 4.1 ± 2.2 |  |
| etoricoxib |  | 0.841 |
| etoricoxib group | 4.5 ± 2.2 |  |
| metamizole group | 4.4 ± 2.1 |  |
| ASA-Status |  | 0.699 |
| I | 4.4 ± 2.3 |  |
| II and III | 4.6 ± 1.9 |  |
| CRP-value |  | 0.668 |
| ≤median | 4.6 ± 2.3 |  |
| >median | 4.4 ± 1.9 |  |

ASA = American Society of Anesthesiologists, CRP = C-reactive protein, SD = standard deviation.
