# Supplementary material for: Pain on the first postoperative day after tonsillectomy in adults: A comparison of metamizole versus etoricoxib as baseline analgesic
Source: PLoS One. 2019 Aug 14;14(8):e0221188. doi: 10.1371/journal.pone.0221188 (PMC6693748; doi:10.1371/journal.pone.0221188)
Supplement: S2 Table — (DOCX) [file pone.0221188.s002.docx]

**S2 Table** Influence of demographic parameters on maximum pain

| Parameter | Mean ± SD | p-value |
| --- | --- | --- |
| maximal pain | 5.8 ± 2.1 |  |
| age |  | **0.049** |
| ≤median | 6.2 ± 2.0 |  |
| >median | 5.4 ± 2.1 |  |
| gender |  | 0.195 |
| female | 6.0 ± 2.2 |  |
| male | 5.6 ± 1.9 |  |
| diagnosis |  | 0.051 |
| chronic tonsillitis | 6.1 ± 2.1 |  |
| peritonsillar abscess | 5.4 ± 2.0 |  |
| etoricoxib |  | 0.267 |
| etoricoxib group | 6.1 ± 1.9 |  |
| metamizole group | 5.6 ± 2.2 |  |
| ASA-Status |  | 0.716 |
| I | 5.9 ± 2.0 |  |
| II and III | 5.7 ± 2.1 |  |
| CRP-value |  | 0.270 |
| ≤median | 6.0 ± 2.2 |  |
| >median | 5.7 ± 2.0 |  |

ASA = American Society of Anesthesiologists, CRP = C-reactive protein, SD = standard deviation.
