# Supplementary material for: Pain on the first postoperative day after tonsillectomy in adults: A comparison of metamizole versus etoricoxib as baseline analgesic
Source: PLoS One. 2019 Aug 14;14(8):e0221188. doi: 10.1371/journal.pone.0221188 (PMC6693748; doi:10.1371/journal.pone.0221188)
Supplement: S3 Table — (DOCX) [file pone.0221188.s003.docx]

**S3 Table** Influence of demographic parameters on minimum pain

| Parameter | Mean ± SD | p-value |
| --- | --- | --- |
| minimal pain | 2.3 ± 1.6 |  |
| age |  | 0.508 |
| ≤median | 2.4 ± 1.6 |  |
| >median | 2.2 ± 1.6 |  |
| gender |  | **0.041** |
| female | 2.6 ± 1.8 |  |
| male | 1.9 ± 1.3 |  |
| diagnosis |  | 0.076 |
| chronic tonsillitis | 2.5 ± 1.8 |  |
| peritonsillar abscess | 1.9 ± 1.2 |  |
| etoricoxib |  | 0.994 |
| etoricoxib group | 2.4 ± 1.7 |  |
| metamizole group | 2.2 ± 1.6 |  |
| ASA-Status |  | **0.019** |
| I | 2.1 ± 1.9 |  |
| II and III | 2.5 ± 1.4 |  |
| CRP-value |  | 0.781 |
| ≤median | 2.3 ± 1.7 |  |
| >median | 2.3 ± 1.6 |  |

ASA = American Society of Anesthesiologists, CRP = C-reactive protein, SD = standard deviation.
