# Supplementary material for: Pain on the first postoperative day after tonsillectomy in adults: A comparison of metamizole versus etoricoxib as baseline analgesic
Source: PLoS One. 2019 Aug 14;14(8):e0221188. doi: 10.1371/journal.pone.0221188 (PMC6693748; doi:10.1371/journal.pone.0221188)
Supplement: S4 Table — (DOCX) [file pone.0221188.s004.docx]

**S4 Table** Influence of demographic parameter on postoperative pain-associated and pain therapy-associated impairments

| Parameter | n | p-value |
| --- | --- | --- |
| impairment in mobility | 16 |  |
| age |  | 0.592 |
| ≤median | 9 |  |
| >median | 7 |  |
| gender |  | 0.945 |
| female | 8 |  |
| male | 8 |  |
| diagnosis |  | 0.657 |
| chronic tonsillitis | 9 |  |
| peritonsillar abscess | 7 |  |
| etoricoxib |  | 0.958 |
| etoricoxib group | 7 |  |
| metamizole group | 9 |  |
| ASA-Status |  | 0.795 |
| I | 7 |  |
| II and III | 9 |  |
| CRP-value |  | 0.971 |
| ≤median | 7 |  |
| >median | 7 |  |
| respiratory impairment | 87 |  |
| age |  | 0.169 |
| ≤median | 40 |  |
| >median | 47 |  |
| gender |  | 0.387 |
| female | 42 |  |
| male | 45 |  |
| diagnosis |  | 0.897 |
| chronic tonsillitis | 53 |  |
| peritonsillar abscess | 34 |  |
| etoricoxib |  | 0.306 |
| etoricoxib group | 36 |  |
| metamizole group | 51 |  |
| ASA-Status |  | **0.008** |
| I | 34 |  |
| II and III | 53 |  |
| CRP-value |  | 0.635 |
| ≤median | 40 |  |
| >median | 37 |  |
| Waking up at night | 66 |  |
| age |  | 0.072 |
| ≤median | 28 |  |
| >median | 38 |  |
| gender |  | 0.866 |
| female | 34 |  |
| male | 32 |  |
| diagnosis |  | **0.016** |
| chronic tonsillitis | 47 |  |
| peritonsillar abscess | 19 |  |
| etoricoxib |  | 0.087 |
| etoricoxib group | 34 |  |
| metamizole group | 32 |  |
| ASA-Status |  | 0.684 |
| I | 32 |  |
| II and III | 34 |  |
| CRP-value |  | **0.018** |
| ≤median | 35 |  |
| >median | 22 |  |
| feeling uncomfortable because of pain | 37 |  |
| age |  | **0.011** |
| ≤median | 12 |  |
| >median | 25 |  |
| gender |  | 0.937 |
| female | 19 |  |
| male | 18 |  |
| diagnosis |  | 0.897 |
| chronic tonsillitis | 23 |  |
| peritonsillar abscess | 14 |  |
| etoricoxib |  | 0.871 |
| etoricoxib group | 16 |  |
| metamizole group | 21 |  |
| ASA-Status |  | 0.785 |
| I | 18 |  |
| I and III | 19 |  |
| CRP-value |  | 0.727 |
| ≤median | 18 |  |
| >median | 16 |  |
| desire for pain killers | 20 |  |
| Age |  | **0.046** |
| ≤median | 6 |  |
| >median | 14 |  |
| gender |  | 0.273 |
| female | 8 |  |
| male | 12 |  |
| diagnosis |  | 0.409 |
| chronic tonsillitis | 14 |  |
| peritonsillar abscess | 6 |  |
| etoricoxib |  | 0.548 |
| etoricoxib group | 10 |  |
| metamizole group | 10 |  |
| ASA |  | 0.534 |
| I | 8 |  |
| II and III | 12 |  |
| CRP-value |  | 0.240 |
| ≤median | 12 |  |
| >median | 7 |  |
| fatigue | 55 |  |
| age |  | 0.643 |
| ≤median | 29 |  |
| >median | 26 |  |
| gender |  | 0.121 |
| female | 32 |  |
| male | 23 |  |
| diagnosis |  | 0.097 |
| chronic tonsillitis | 38 |  |
| peritonsillar abscess | 17 |  |
| etoricoxib |  | 0.675 |
| etoricoxib group | 23 |  |
| metamizole group | 32 |  |
| ASA-Status |  | 0.286 |
| I | 23 |  |
| II and III | 32 |  |
| CRP-value |  | 0.565 |
| ≤median | 26 |  |
| >median | 23 |  |
| nausea | 7 |  |
| age |  | 1.000 |
| ≤median | 3 |  |
| >median | 4 |  |
| gender |  | 0.440 |
| female | 5 |  |
| male | 2 |  |
| diagnosis |  | **0.042** |
| chronic tonsillitis | 7 |  |
| peritonsillar abscess | 0 |  |
| etoricoxib |  | 0.699 |
| etoricoxib group | 4 |  |
| metamizole group | 3 |  |
| ASA-Status |  | 0.050 |
| I | 6 |  |
| II and III | 1 |  |
| CRP-value |  | 0.206 |
| ≤median | 5 |  |
| >median | 1 |  |
| vomitus | 4 |  |
| age |  | 1.000 |
| ≤median | 2 |  |
| >median | 2 |  |
| gender |  | 0.619 |
| female | 3 |  |
| male | 1 |  |
| diagnosis |  | 0.158 |
| chronic tonsillitis | 4 |  |
| peritonsillar abscess | 0 |  |
| etoricoxib |  | 1.000 |
| etoricoxib group | 2 |  |
| metamizole group | 2 |  |
| ASA-Status |  | 0.339 |
| I | 3 |  |
| II and III | 1 |  |
| CRP-value |  | 0.118 |
| ≤median | 4 |  |
| >median | 0 |  |

ASA = American Society of Anesthesiologists, CRP = C-reactive protein, SD = standard deviation.
