# Supplementary material for: Pain on the first postoperative day after tonsillectomy in adults: A comparison of metamizole versus etoricoxib as baseline analgesic
Source: PLoS One. 2019 Aug 14;14(8):e0221188. doi: 10.1371/journal.pone.0221188 (PMC6693748; doi:10.1371/journal.pone.0221188)
Supplement: S5 Table — (DOCX) [file pone.0221188.s005.docx]

**S5 Table** Influence of demographic parameters on the satisfaction with pain therapy

| Parameter | Mean ± SD | p-value |
| --- | --- | --- |
| satisfaction | 11.6 ± 3.2 |  |
| age |  | **0.006** |
| ≤median | 11.1 ± 3.0 |  |
| >median | 12.1 ± 3.3 |  |
| gender |  | 0.910 |
| female | 11.4 ± 3.7 |  |
| male | 11.8 ± 2.7 |  |
| diagnosis |  | **0.002** |
| chronic tonsillitis | 11.1 ± 3.3 |  |
| peritonsillar abscess | 12.5 ± 2.9 |  |
| etoricoxib |  | 0.916 |
| etoricoxib group | 11.7 ± 3.0 |  |
| metamizole group | 11.5 ± 3.4 |  |
| ASA-Status |  | 0.784 |
| I | 11.4 ± 3.6 |  |
| II and III | 11.8 ± 2.9 |  |
| CRP-value |  | 0.365 |
| ≤median | 11.2 ± 3.5 |  |
| >median | 11.8 ± 3.1 |  |

ASA = American Society of Anesthesiologists, CRP = C-reactive protein, SD = standard deviation.
