# Supplementary material for: Pain on the first postoperative day after tonsillectomy in adults: A comparison of metamizole versus etoricoxib as baseline analgesic
Source: PLoS One. 2019 Aug 14;14(8):e0221188. doi: 10.1371/journal.pone.0221188 (PMC6693748; doi:10.1371/journal.pone.0221188)
Supplement: S6 Table — (DOCX) [file pone.0221188.s006.docx]

**S6 Table** Influence of process parameter on pain in activity

| Parameter | Mean ± SD | p-value |
| --- | --- | --- |
| pain in activity | 4.5 ± 2.1 |  |
| sedativum |  | 0.259 |
| no | 5.1 ± 1.7 |  |
| midazolam | 4.4 ± 2.1 |  |
| preoperative etoricoxib |  | 0.326 |
| yes | 4.6 ± 2.1 |  |
| no | 3.5 ± 2.0 |  |
| intraoperative remifentanil |  | 0.217 |
| yes | 4.2 ± 2.0 |  |
| no | 4.8 ± 2.2 |  |
| intraoperative clonidine |  | 0.442 |
| yes | 4.9 ± 2.2 |  |
| no | 4.4 ± 2.1 |  |
| opioids in recovery room |  | 0.169 |
| yes | 4.8 ± 2.0 |  |
| no | 4.2 ± 2.2 |  |
| dominant opioid in recovery room |  | 0.441 |
| piritramide | 4.8 ± 2.0 |  |
| pethidine | 3.0 |  |
| metamizole in recovery room |  | 0.099 |
| yes | 4.8 ± 2.0 |  |
| no | 4.2 ± 2.1 |  |
| opioids on ward |  | **0.001** |
| yes | 4.9 ± 2.2 |  |
| no | 3.6 ± 1.6 |  |
| tramadol dominant on ward |  | 0.642 |
| yes | 4.8 ± 2.0 |  |
| no | 4.4 ± 2.2 |  |
| piritramide dominant on ward |  | **0.005** |
| yes | 4.9 ± 2.2 |  |
| no | 3.8 ± 1.8 |  |
| tilidine dominant on ward |  | 0.306 |
| yes | 7.0 |  |
| no | 4.4 ± 2.1 |  |
| acetaminophen dominant on ward |  | 0.887 |
| yes | 4.0 |  |
| no | 4.5 ± 2.1 |  |
| metamizole dominant non-opioid on ward |  | 0.859 |
| yes | 4.4 ± 2.1 |  |
| no | 4.5 ± 2.1 |  |
| etoricoxib dominant non-opioid on ward |  | 0.841 |
| yes | 4.5 ± 2.2 |  |
| no | 4.4 ± 2.1 |  |
| additional opioid on ward |  | 0.200 |
| no | 4.4 ± 2.1 |  |
| tramadol | 5.0 ± 1.6 |  |
| metamizole as additional non-opioid on ward |  | 0.060 |
| yes | 5.2 ± 2.4 |  |
| no | 4.3 ± 2.0 |  |
| ibuprofen as additional non-opioid on ward |  | 0.840 |
| yes | 4.6 ± 2.3 |  |
| no | 4.5 ± 2.1 |  |
| preoperative pain therapy |  | **0.003** |
| yes | 5.4 ± 2.1 |  |
| no | 4.2 ± 1.6 |  |
| physical pain therapy |  | 0.528 |
| yes | 5.4 ± 2.1 |  |
| no | 4.2 ± 2.0 |  |
| Individual therapy |  | 0.599 |
| yes | 4.5 ± 2.1 |  |
| no | 5.0 ± 1.7 |  |
| pain documentation |  | 0.547 |
| yes | 4.5 ± 2.1 |  |
| no | 4.2 ± 1.8 |  |
| preoperative counseling on postoperative pain management |  | **0.009** |
| no | 5.1 ± 3.1 |  |
| yes, general | 4.7 ± 2.1 |  |
| yes, special | 3.3 ± 1.6 |  |
