# Supplementary material for: Pain on the first postoperative day after tonsillectomy in adults: A comparison of metamizole versus etoricoxib as baseline analgesic
Source: PLoS One. 2019 Aug 14;14(8):e0221188. doi: 10.1371/journal.pone.0221188 (PMC6693748; doi:10.1371/journal.pone.0221188)
Supplement: S7 Table — (DOCX) [file pone.0221188.s007.docx]

**S7 Table** Influence of process parameter on maximum pain

| Parameter | Mean ± SD | p-value |
| --- | --- | --- |
| E3: maximum pain | 5.8 ± 2.1 |  |
| sedativum |  | 0.986 |
| no | 5.8 ± 1.9 |  |
| midazolam | 5.8 ± 2.1 |  |
| preoperative etoricoxib |  | 0.543 |
| yes | 6.1 ± 1.9 |  |
| no | 5.2 ± 1.9 |  |
| intraoperative remifentanil |  | 0.688 |
| yes | 5.7 ± 2.1 |  |
| no | 5.9 ± 2.0 |  |
| intraoperative clonidine |  | 0.186 |
| yes | 6.4 ± 2.3 |  |
| no | 5.7 ± 2.0 |  |
| opioids in recovery room |  | **0.011** |
| yes | 6.3 ± 1.9 |  |
| no | 5.3 ± 2.1 |  |
| dominant opioid in recovery room |  | 0.847 |
| piritramide | 6.3 ± 1.9 |  |
| pethidine | 6.0 |  |
| metamizole in recovery room |  | 0.572 |
| yes | 5.9 ± 2.2 |  |
| no | 5.8 ± 2.0 |  |
| opioid on ward |  | **<0.001** |
| yes | 6.3 ± 1.9 |  |
| no | 4.7 ± 2.0 |  |
| tramadol dominant opioid on ward |  | 0,518 |
| yes | 6.3 ± 1.9 |  |
| no | 5.8 ± 2.0 |  |
| piritramide dominant opioid on ward |  | **0.001** |
| yes | 6.3 ± 1.9 |  |
| no | 5.0 ± 2.1 |  |
| tilidin dominant opioid on ward |  | 0.081 |
| yes | 9.0 |  |
| no | 5.8 ± 2.0 |  |
| acetaminophen dominant non-opioid on ward |  | 0.732 |
| yes | 5.0 |  |
| no | 5.8 ± 2.1 |  |
| metamizole dominant non-opioid on ward |  | 0.384 |
| yes | 5.6 ± 2.2 |  |
| no | 6.0 ± 1.9 |  |
| etoricoxib dominant non-opioid on ward |  | 0.267 |
| yes | 6.1 ± 1.9 |  |
| no | 5.6 ± 2.2 |  |
| additional opioid on ward |  | 0.060 |
| no | 5.7 ± 2.1 |  |
| tramadol | 6.9 ± 1.7 |  |
| metamizole as additional non-opioid on ward |  | **0.014** |
| yes | 6.7 ± 1.7 |  |
| no | 5.6 ± 2.1 |  |
| ibuprofen as additional non-opioid on ward |  | 0.258 |
| yes | 5.0 ± 2.0 |  |
| no | 5.9 ± 2.1 |  |
| preoperative pain therapy |  | **0.020** |
| yes | 6.5 ± 2.0 |  |
| no | 5.6 ± 2.0 |  |
| physical pain therapy |  | 0.687 |
| yes | 6.5 ± 2.0 |  |
| no | 5.6 ± 2.0 |  |
| individual therapy |  | 0.859 |
| yes | 5.8 ± 2.1 |  |
| no | 6.0 ± 2.6 |  |
| pain documentation |  | 0.461 |
| yes | 5.9 ± 2.0 |  |
| no | 5.4 ± 2.5 |  |
| preoperative counseling on postoperative pain management |  | **0.004** |
| no | 7.2 ± 1.2 |  |
| yes, general | 4.7 ± 2.1 |  |
| yes, special | 3.3 ± 1.6 |  |
