# Supplementary material for: Pain on the first postoperative day after tonsillectomy in adults: A comparison of metamizole versus etoricoxib as baseline analgesic
Source: PLoS One. 2019 Aug 14;14(8):e0221188. doi: 10.1371/journal.pone.0221188 (PMC6693748; doi:10.1371/journal.pone.0221188)
Supplement: S8 Table — (DOCX) [file pone.0221188.s008.docx]

**S8 Table** Influence of process parameter on minimum pain

| Parameter | Mean ± SD | p-value |
| --- | --- | --- |
| minimal pain | 2.3 ± 1.6 |  |
| sedativum |  | 0.416 |
| no | 2.6 ± 1.4 |  |
| midazolam | 2.3 ± 1.6 |  |
| preoperative etoricoxib |  | 0.770 |
| yes | 2.4 ± 1.7 |  |
| no | 1.6 ± 1.6 |  |
| intraoperative remifentanil |  | 0.750 |
| yes | 2.0 ± 1.6 |  |
| no | 2.3 ± 1.6 |  |
| intraoperativ clonidine |  | 0.106 |
| yes | 2.8 ± 1.5 |  |
| no | 2.2 ± 1.6 |  |
| opioids in recovery room |  | 0.870 |
| yes | 2.2 ± 1.6 |  |
| no | 2.3 ± 1.6 |  |
| dominant opioid in recovery room |  | 0.576 |
| piritramide | 2.2 ± 1.6 |  |
| pethidine | 3.0 |  |
| metamizole in recovery room |  | **0.049** |
| yes | 2.6 ± 1.5 |  |
| no | 2.1 ± 1.6 |  |
| opioid on ward |  | 0.881 |
| yes | 2.3 ± 1.7 |  |
| no | 2.2 ± 1.5 |  |
| tramadol dominant opioid on ward |  | 0.798 |
| yes | 2.3 ± 1.6 |  |
| no | 2.3 ± 1.6 |  |
| piritramid dominant opioid on ward |  | 0.811 |
| yes | 2.3 ± 1.7 |  |
| no | 2.3 ± 1.5 |  |
| tilidin dominant opioid on ward |  | 0.177 |
| yes | 5.0 |  |
| no | 2.3 ± 1.6 |  |
| acetaminophen dominant non-opioid on ward |  | 0.565 |
| yes | 3.0 |  |
| no | 2.3 ± 1.6 |  |
| metamizole dominant non-opioid on ward |  | 0.774 |
| yes | 2.2 ± 1.6 |  |
| no | 2.4 ± 1.7 |  |
| etoricoxib dominant non-opioid on ward |  | 0.994 |
| yes | 2.4 ± 1.7 |  |
| no | 2.2 ± 1.6 |  |
| additional opioid on ward |  | 0.890 |
| no | 2.3 ± 1.7 |  |
| tramadol | 1.9 ± 1.1 |  |
| metamizole as additional non-opioid on ward |  | 0.143 |
| yes | 2.7 ± 1.8 |  |
| no | 2.2 ± 1.6 |  |
| ibuprofen as additional non-opioid on ward |  | 0.715 |
| yes | 2.6 ± 2.1 |  |
| no | 2.3 ± 1.6 |  |
| preoperative pain therapy |  | 0.304 |
| yes | 2.5 ± 1.5 |  |
| no | 2.2 ± 1.6 |  |
| physical pain therapy |  | 0.108 |
| yes | 2.5 ± 1.5 |  |
| no | 2.2 ± 1.6 |  |
| individual therapy |  | 0.689 |
| yes | 2.3 ± 1.6 |  |
| no | 2.3 ± 0.6 |  |
| pain documentation |  | 0.311 |
| yes | 2.3 ± 1.7 |  |
| no | 2.5 ± 1.4 |  |
| preoperative counseling on postoperative pain management |  | 0.280 |
| no | 2.2 ± 1.6 |  |
| yes, general | 2.4 ± 1.7 |  |
| yes, special | 1.8 ± 1.3 |  |
